# Helix α-3 inter-molecular salt bridges and conformational changes are essential for toxicity of *Bacillus thuringiensis* 3D-Cry toxin family

**DOI:** 10.1038/s41598-018-28753-8

**Published:** 2018-07-09

**Authors:** Sabino Pacheco, Isabel Gómez, Jorge Sánchez, Blanca-Ines García-Gómez, Daniel M. Czajkowsky, Jie Zhang, Mario Soberón, Alejandra Bravo

**Affiliations:** 1 Instituto de Biotecnología, Universidad Nacional Autónoma de México, Apdo. Postal 510-3, Cuernavaca, 62250 Morelos, Mexico; 2 Bio-ID Center, School of Biomedical Engineering, Shanghai, 200240 China; 3 State Key Laboratory for Biology of Plant Diseases and Insect Pests, Institute of Plant Protection, Chinese Academy of Agricultural Sciences, Beijing, 100193 P. R. China

## Abstract

*Bacillus thuringiensis* insecticidal Cry toxins break down larval midgut-cells after forming pores. The 3D-structures of Cry4Ba and Cry5Ba revealed a trimeric-oligomer after cleavage of helices α-1 and α-2a, where helix α-3 is extended and made contacts with adjacent monomers. Molecular dynamic simulations of Cry1Ab-oligomer model based on Cry4Ba-coordinates showed that E101 forms a salt-bridge with R99 from neighbor monomer. An additional salt bridge was identified in the trimeric-Cry5Ba, located at the extended helix α-3 in the region corresponding to the α-2b and α-3 loop. Both salt-bridges were analyzed by site directed mutagenesis. Single-point mutations in the Lepidoptera-specific Cry1Ab and Cry1Fa toxins were affected in toxicity, while reversed double-point mutant partially recovered the phenotype, consistent with a critical role of these salt-bridges. The single-point mutations in the salt-bridge at the extended helix α-3 of the nematicidal Cry5Ba were also non-toxic. The incorporation of this additional salt bridge into the nontoxic Cry1Ab-R99E mutant partially restored oligomerization and toxicity, supporting that the loop between α-2b and α-3 forms part of an extended helix α-3 upon oligomerization of Cry1 toxins. Overall, these results highlight the role in toxicity of salt-bridge formation between helices α-3 of adjacent monomers supporting a conformational change in helix α-3.

## Introduction

Cry toxins produced by *Bacillus thuringiensis* (Bt) are toxic to diverse insect species and other invertebrates and have been used to control insect pests in agriculture and against dipteran insects that are vectors of human diseases^1^.

The three-domain Cry (3d-Cry) toxin family is a large protein family with many members showing high specificity against different insect orders^2^. Some of these proteins have been successfully expressed in plants such as Cry1Ac in cotton or Cry1Ab and Cry1Fa in maize, resulting in crop protection from insect attack with an important reduction in the utilization of chemical insecticides and a significant increase in crop yields in certain countries^3^.

The three-dimensional structure of nine 3d-Cry proteins shows a similar structural fold composed of three domains, suggesting a similar mode of action of the members of this protein family^4–11^. Domain I, composed of a seven α-helix bundle, is involved in oligomerization and pore formation, while domains II and III are mainly composed of β-sheets and are involved in recognition of membrane proteins in the larval midgut cells and thus are essential for conferring toxin specificity^1^.

The model of the mechanism of action of 3d-Cry toxin that is more accepted and has more experimental support proposes that 3d-Cry are pore-forming toxins that exert their toxic effect by forming pores in the insect gut cells leading to osmotic shock, cell burst and death of the larvae. Cry1A toxins are produced as 130 kDa protoxins that are solubilized in the midgut and activated by proteases, resulting in a 60 kDa protease resistant core composed of the three structural domains^1^. The Cry1A proteins undergo sequential binding interactions with different insect midgut proteins including cadherin (CAD) and glycosyl-phosphatidyl-inositol (GPI)-anchored proteins such as alkaline phosphatase (ALP) or aminopeptidase N (APN)^12^. The interaction of Cry proteins with the transmembrane CAD protein plays a fundament role in inducing the formation of an oligomeric structure that inserts into the membrane to form the pore^12–18^. For oligomerization, it was proposed that the amino terminal end including helix α-1 is cleaved out^19^. The Cry1Ab modified protein (Cry1AMod), with the amino terminal end deleted including helix α-1 and part of helix α-2a, is capable of forming oligomeric structures in the absence of CAD and kills insects that are resistant due to mutations linked to CAD and other receptors^20,21^. Toxins with mutations in helix α-3, such as R99E in Cry1Ab, were affected in oligomerization and toxicity to *Manduca sexta* larvae^22^. Similarly, Cry11Aa toxin helix α-3 mutants were also defective in oligomerization and toxicity to *Aedes aegypti* larvae^23^. Analysis *in silico* and equilibrium sedimentation data of helix α-3 of domain I of Cry1A toxin showed that this region has homo-oligomerization tendencies and supports that R99 residue from Cry1A helix α-3 participates in Cry toxin oligomerization^24^. However, the oligomerization of 3d-Cry toxins has been studied only in a limited number of toxins (Cry1A, Cry3Aa, Cry4Ba and Cry11Aa). It was shown that similar to the Cry1A toxins, the Cry11Aa and Cry3Aa toxins also require binding to CAD to oligomerize^25–27^. In contrast the Cry4Ba toxin is an exception because it is able to oligomerize *in vitro* in the absence of CAD binding^27,28^.

**Figure 1:**
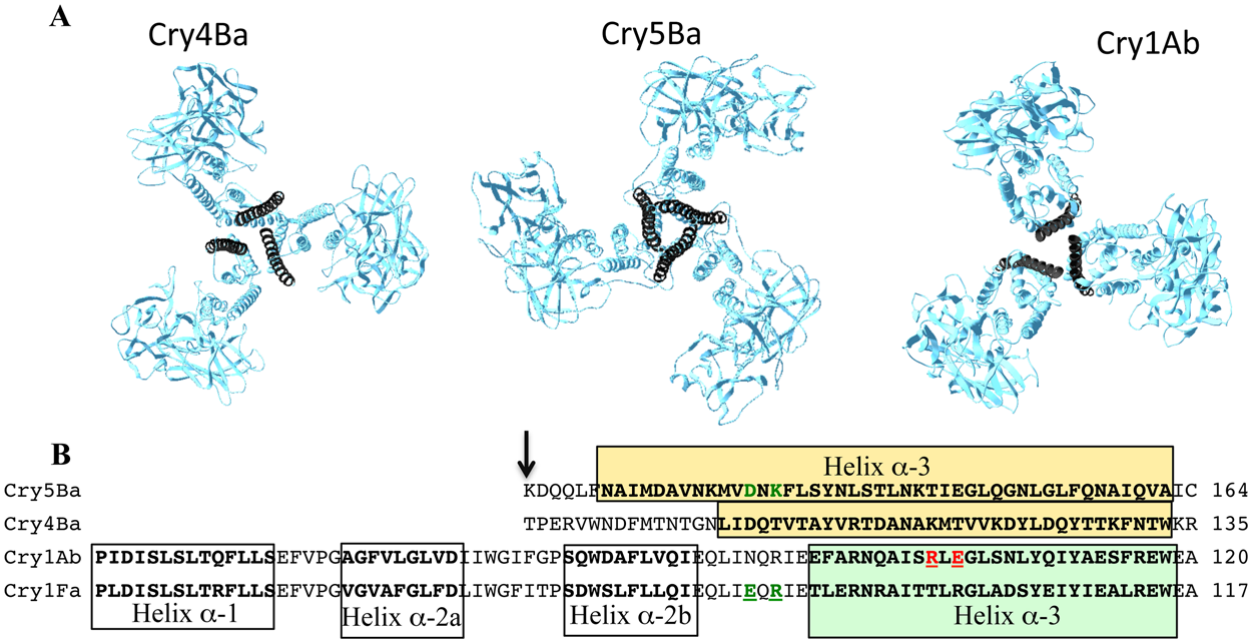
Crystal structure of the trimeric organization of Cry4Ba and Cry5Ba and the model of the Cry1Ab trimer. Panel A, Trimeric organization of the three dimensional structure of Cry4Ba and Cry5Ba toxins (pdb:1W99 and 4D8M). These structures were obtained from truncated proteins where helices α-1 and α-2a were lost. The extended helix α-3 is presented in black color. The model of the trimeric organization of Cry1Ab was constructed using Pymol using the Cry4Ba-trimer as a template. The coordinates of the trimeric Cry1Ab structure will be available in Dryad (https://datadryad.org) once the paper is published. Panel B, Alignment of N-terminal amino acid sequences of Cry4Ba and Cry5Ba toxins that were resolved by X-ray diffraction and comparison with the primary toxin sequences of Cry1Ab and Cry1Fa. The amino acid sequence of helix α-3 is shown inside the yellow and green boxes in the different toxins. The first salt bridge localized in Cry1Ab is presented in red letters and the second salt bridge localized in Cry5Ba and Cry1Fa is presented in green letters.

The crystal structures of Cry4Ba and Cry5Ba toxins had previously been obtained by other groups, revealing a trimeric organization where helices α-1 and α-2a were lost during the crystallization process (Fig. 1A) (pdb: 1W99 and 4D8M)^8,11^. It is remarkable that the cleavage of both proteins Cry4Ba and Cry5Ba was equivalent, located 50 residues upstream of the end of helix α-3 (Fig. 1B). In addition, these structures show a conformational change in the structure of helix α-2b and the loop connecting to helix α-3 that resulted in its arrangement forming an extended long helix α-3 in the trimer (Fig. 1B). The long α-3 helices of the Cry4Ba and Cry5Ba toxin structures are important for their trimeric organization since this long α-3 helix makes several contacts with other helices (α-3, α-4 and α-6) from the adjacent monomers^8,11^. In addition, Cry1AbMod inserted in synthetic membranes showed a trimeric organization in 2D crystals observed by electron microscopy^29^. Similarly the Cry4Ba toxin showed a trimeric organization after incorporation into synthetic membrane observed by electron microscopy^28^. The trimeric 3D structure of Cry4Ba was used to construct a model of the trimeric structure of Cry4Aa and the authors hypothesized that this structure may resemble the oligomer structure involved in pore formation and toxicity of Cry4Aa^30^.

To further analyze the role of different residues of Cry1Ab helix α-3 in oligomerization, a structural-model of the Cry1Ab trimeric oligomer based on the coordinates of the Cry4Ba trimeric structure was constructed. We found that a glutamic residue (E101) located in α-3 helix was in close proximity to R99 from the adjacent monomer suggesting that these two residues may form a salt bridge between adjacent monomers. This putative salt bridge is conserved in part of the 3d-Cry toxin family since it was found in 24 out of 91 representative 3d-Cry sequences. Sequence alignment of the 3d-Cry family revealed an additional putative salt bridge that is present in 48 out of 91 3d-Cry sequences with a similar orientation as the salt bridge formed by R99-E101. This salt-bridge is conserved in Cry5Ba and it is present in the reported trimer structure (pdb: 4D8M). The role of these two salt bridges on Cry toxin action was confirmed by an examination of single point mutations as well as double-point mutants with reversed charges in different 3d-Cry toxins. Overall the results presented here show that the contacts between helices α-3 of different monomers have an important role in the toxicity of 3d-Cry toxins, and that oligomer formation is a key step in the mode of action of these insecticidal proteins.

## Results

### Identification of a putative salt bridge in the Cry1Ab trimer

**Figure 2:**
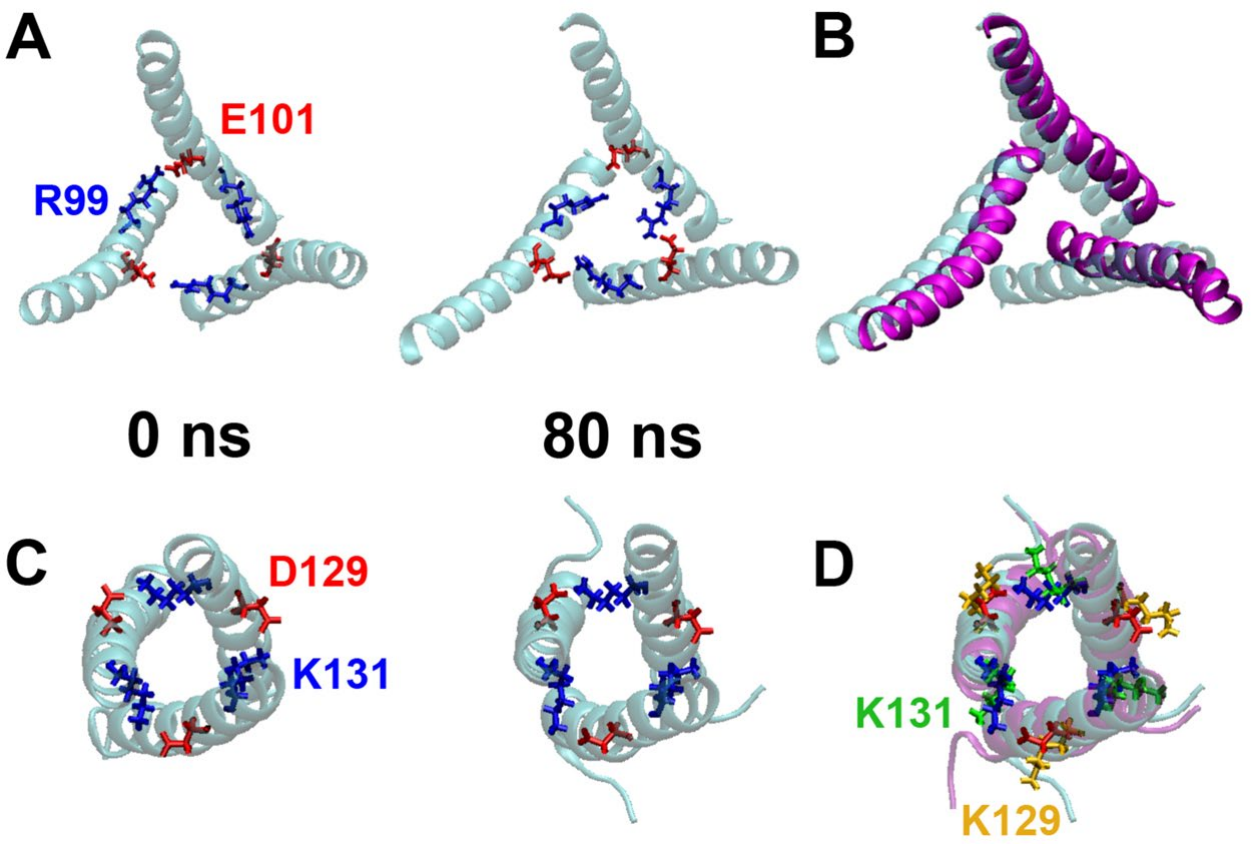
Analysis of salt bridges by MD simulations. Panel A, Identification of an inter-molecular salt bridge between R99 and E101 of Cry1Ab in the homology model of the trimer. The left panel is the structure at the start of the simulations and the right panel is the structure at the end of the simulations. These salt bridges remain intact throughout the simulations. The helix α-3 is shown in blue. Panel B, The single Cry1Ab-R99E mutation leads to a collapse of the local α-3 helical organization and a loss of the three-fold symmetry present in the wild-type trimer. Shown in purple are the α-3 helices in the Cry1Ab-R99E mutant at the end of the simulations overlapping those of the wild-type protein. Panel C, Identification of an inter-molecular salt bridge between D129 and K131 of Cry5Ba in the crystal structure of the trimer at the start and end of the simulations. These salt bridges are also present throughout the simulations. The helix α-3 is shown in blue. Panel D, Although the local helical structure of the Cry5Ba-D129K mutant toxin remains unchanged from that of the wild-type protein, it is clear that the K129 residues re-orient away from the neighboring K131 residues during these simulations, in contrast with the D129 residues in the wild-type protein. The helix α-3 of the mutant protein is colored purple.

A structural-model of the Cry1Ab trimer (Fig. 1A) was obtained based on the coordinates of the Cry4Ba trimeric structure (pdb: 1W99) as described in methods. In this model, the arginine residue, R99, in the α-3 helix was observed to be in close proximity to a glutamic acid residue, E101, in the α-3 helix of an adjacent toxin-monomer (Fig. 2A). Extended molecular dynamic (MD) simulations showed that these residues were indeed involved in an inter-molecular salt bridge that remained largely interacting throughout the simulations, suggesting that this interaction could be important for oligomer stability (Fig. 2A). Consistent with this, simulations of the mutant R99E revealed a rapid collapse of the local organization of the α-3 helices and a striking loss of the three-fold symmetry that is present throughout the simulations of the wild-type protein (Fig. 2B). Such a significant structural change from only a single mutation strongly suggests that this salt bridge may play an important role in stabilizing the oligomeric architecture of this toxin.

### Identification of a conserved additional salt bridge among different Cry toxins

**Figure 3:**
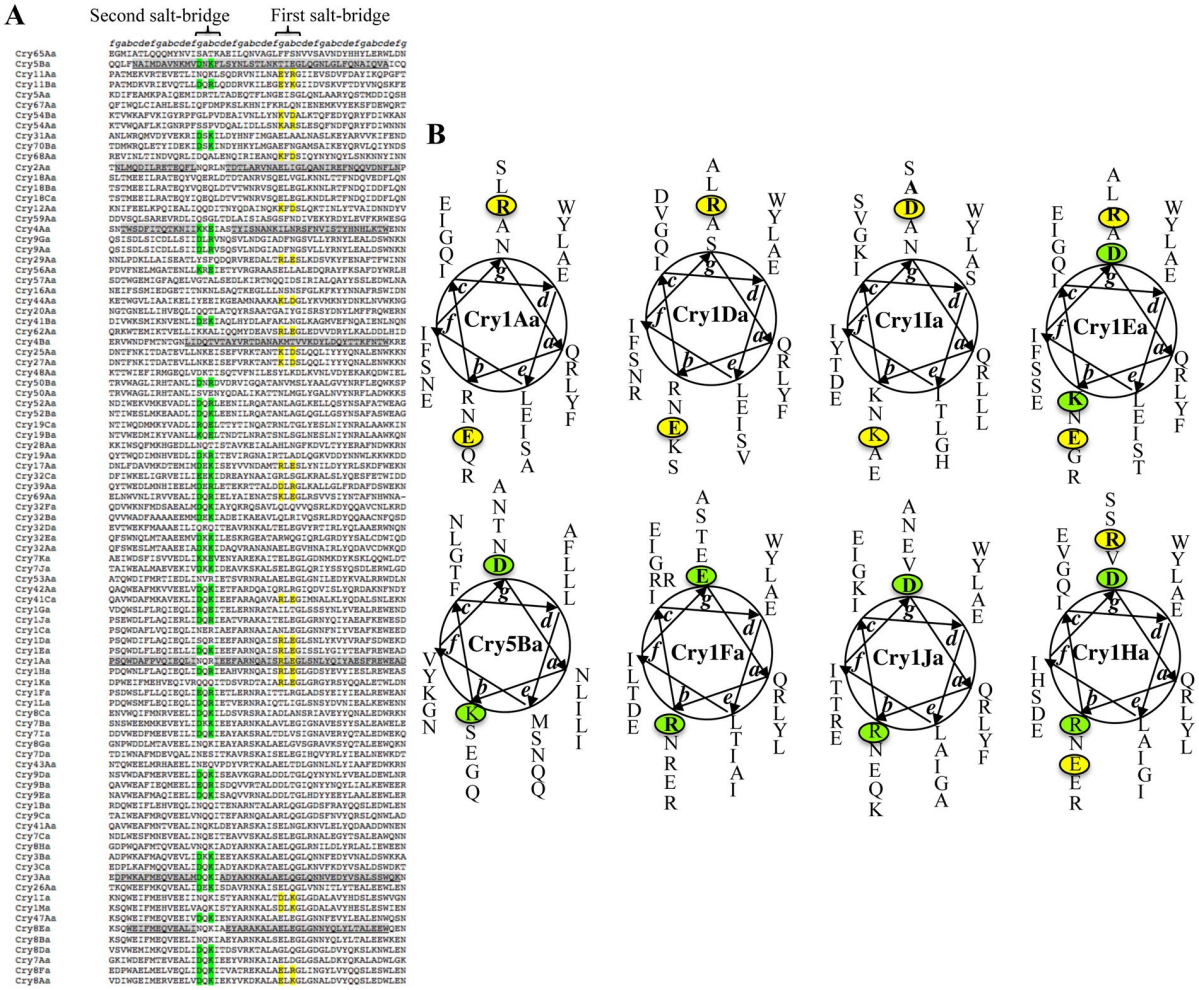
Localization of two putative salt bridges in the primary sequence of 3d-Cry toxin proteins. Panel A, alignment of amino acid sequences of helices α-2 and α-3 from of 91 different 3d-Cry toxins including one representative of each 3d-Cry type (with the subindex *a* in the third range of the nomenclature). The putative salt bridges between two different monomers are shown in yellow or in green letters. The sequences of helices α-2 and α-3 in the different proteins where the structure was solved are underlined and shown in grey color. Panel B, schematic representation of the predicted extended helix α-3 region from different representative Cry toxins. The residues involved in a salt bridge between different monomers are encircled, these residues are located in two opposite faces of the putative helix α-3.

We aligned Cry toxins from the 3d-Cry family (we aligned one representative Cry member of each subgroup) to determine if the potential salt bridge, R99-E101, of Cry1Ab is conserved in this group of proteins. Figure 3A shows that this putative salt bridge is present in a representative number of toxins (24 out of 91 3d-Cry sequences, yellow letters). However, inspection of the sequence of helix α-3 from this protein family revealed an alternative pair of charged residues that are located in the same “faces” of the α-3 helix as the R99-E101 pair (Fig. 3B). This second putative salt bridge is more conserved in the 3d-Cry family (48 out of 91 Cry toxin sequences) including the nematicidal Cry5Ba toxin (Fig. 3A, green letters). Structural analysis of Cry5Ba showed that residues Cry5Ba-D129 and Cry5Ba-K131 are located in the extended helix α-3 forming a salt bridge between different monomers in the trimeric crystal structure (Figs 1B and 2C). It is interesting to note that these residues are located in the loop between helices α2b and α3 in the family of 3d-Cry toxins but that in the Cry5Ba and Cry4Ba trimeric structures, they form part of the extended helix α-3 (Fig. 1B, green letters). MD simulations performed with a D129K mutant of Cry5Ba showed that this mutation is likely to affect the stability of the trimer, since the K129 residue rapidly re-orients away from K131 during the simulations and remains in this distant orientation throughout the duration of the simulations (Fig. 2D).

### Construction of single and double mutants

A useful strategy that has been used to demonstrate that two charged residues participate in a salt bridge is to make single-point mutants that affect the protein activity and a double-point mutant with reversed charges that restored such activity^31–33^. We decided to use this strategy to determine the role of these potential salt bridges in the toxicity of Cry toxins. Single-point and double-point mutants with the reversed charges were constructed and characterized. To study the first salt bridge, a Cry1Ab-E101K single-point mutant and a double-point mutant Cry1Ab-R99E-E101R were constructed. In the case of the second salt bridge, mutants in Cry1Fa and Cry5Ba were constructed, including single-point mutants, Cry1Fa-E83R, Cry1Fa-R85E, Cry5Ba D129K and Cry5Ba-K131D and double-point mutants, Cry1Fa-E83R-R85E and Cry5Ba D129K-K131D.

**Figure 4:**
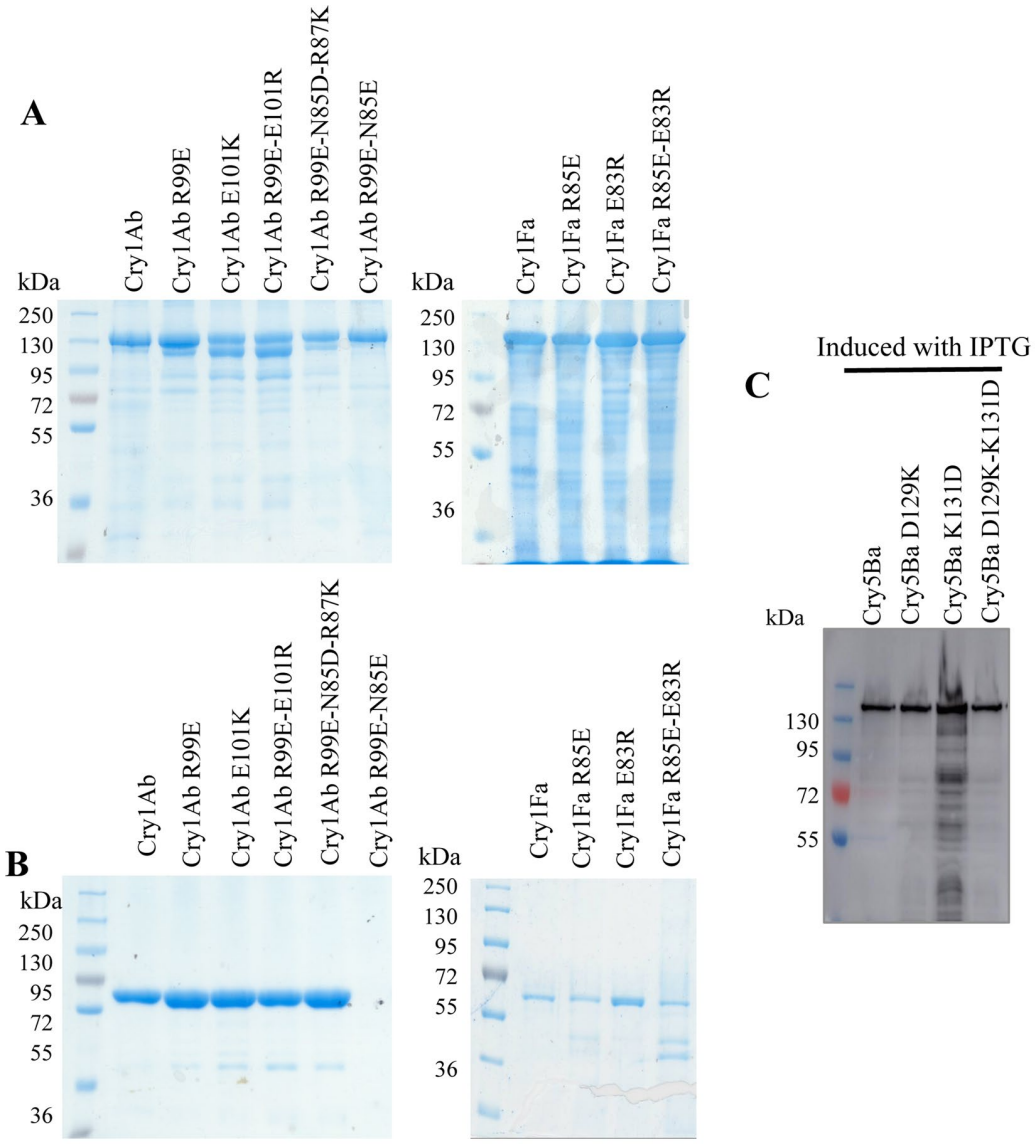
Analysis of the expression of different Cry1Ab, Cry1F mutants in Bt cells and Cry5Ba mutants in *E. coli* cells. Panel A, SDS-PAGE electrophoresis of Cry1Ab and Cry5Ba protoxins. Panel B, SDS-PAGE electrophoresis of trypsin-activated Cry1Ab and Cry1Fa toxins. Panel C, Western blot analysis of the expression of Cry5Ba in *E. coli* cells. All samples were boiled 5 min in Laemmli sample buffer before loading into the SDS-PAGE.

Cry1Ab and Cry1Fa wild type and mutant proteins were expressed in Bt transformant strains, parasporal crystals were solubilized in alkaline pH and processed by trypsin treatment as described in Methods. In the case of the nematicidal Cry5Ba and mutants, these proteins were produced in *Escherichia coli* and their expression was induced with 50 μM isopropyl β-D-thiogalactoside as previously described to perform bioassays against the nematode *Caenorhabditis elegans* with intact *E. coli* cells expressing these proteins^34^. Figure 4A shows that all Cry1Ab and Cry1Fa mutant proteins are produced as 130 kDa protoxins, that after activation with trypsin resulted in a 60 kDa proteins similar to the wild type Cry1Ab and Cry1Fa toxins (Fig. 4B), suggesting that these mutations did not alter drastically the structure of the monomeric toxins. In the case of Cry5Ba proteins, the expression of a 140 kDa protein was confirmed by western blot as described in the Methods showing that the three mutants have similar expression levels as Cry5Ba (Fig. 4C).

### Effects of single mutations

To determine the effects of the single point mutations on the toxicity of Cry1Ab and Cry1Fa toxins, bioassays were conducted with two lepidopteran insects, *Plutella xylostella* and *M. sexta* that are sensitive to both toxins. Table 1 shows that Cry1Ab-R99E mutant was not toxic as previously reported^22^. Cry1Ab-E101K mutation also was not toxic to *P. xylostella* and *M. sexta* since no mortality was observed even when they were exposed at the highest toxin concentration (10,000 ng/cm^2^). In contrast, the wild type Cry1Ab showed an LC_50_ value of 2.3 and 2.4 ng/cm^2^ in *P. xylostella* and in *M. sexta*, respectively. Single-point Cry1Fa mutants, Cry1Fa-E83R and Cry1Fa-R85E, were also affected in toxicity, showing a reduction of 9- and 7-fold in their potency against *P. xylostella* and 15- and 13- fold lower toxicity against *M. sexta*, when compared with the Cry1Fa toxin (Table 1). The Cry5Ba toxicity was analyzed in bioassays with *C. elegans* nematodes that were fed with *E. coli* cells expressing the different proteins. Nematode bioassays performed with the Cry5Ba single point mutations D129K and K131D showed no toxicity to *C. elegans* in contrast to Cry5Ba (Dr. Raffi Aroian personal communication).

**Table 1:** Bioassays against *Plutella xylostella* and *Manduca sexta* larvae.

Protein	*Plutella xylostella* ^b^LC_50_ (fiducial limits)	Potency loss^a^ Fold	*Manduca sexta* ^b^LC_50_ (fiducial limits)	Potency loss^a^ Fold
Cry1Ab	2.3 (1.9–2.5)	—	2.4 (1.5–3.2)	—
Cry1Ab R99E	>10,000	>4,347	>10,000	>4,166
Cry1Ab E101K	>10,000	>4,347	>10,000	>4,166
Cry1Ab R99E-E101R	162.1 (123.1–205.0)	70	278.6 (218.7–362.5)	116
Cry1Ab-R99E-N85D-R87K	215 (182.1–259.1)	93	250.6 (90.9–361.8)	104
Cry1Fa	2.2 (1.5–2.8)	—	2.0 (1.1–2.7)	—
Cry1Fa E83R	20.0 (16.5–24.1)	9	29.8 (21.1–39.4)	14.9
Cry1Fa R85E	15.9 (11.0–19.5)	7	26.8 (21.5–33.2)	13.2
Cry1Fa E83R-R85E	6.5 (4.2–8.2)	2.8	9.2 (6.6–11.6)	4.6

^a^Potency loss corresponds to LC_50_ mutant/LC_50_ wild type.

^b^LC_50_ value in ng toxin/cm^2^ diet.

**Figure 5:**
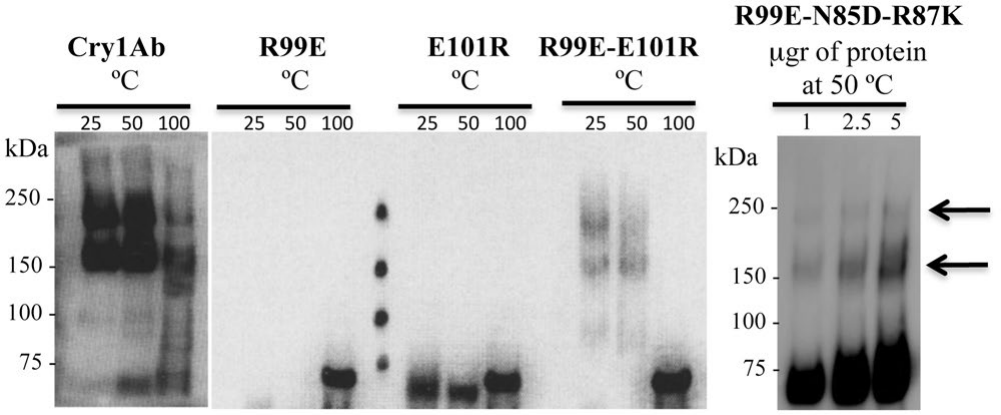
Oligomer formation of Cry1Ab toxins. Oligomerization of Cry1Ab wild type and mutant toxins after incubation with a CAD fragment from *M. sexta* and trypsin for 30 min, samples were heated 3 min at 25 °C, 50 °C or 100 °C before running the samples on SDS-PAGE electrophoresis and oligomers were revealed by western blot with an anti-Cry1Ab polyclonal antibody. Full-length blots are included in supplementary information figure S1.

It was previously reported that Cry1Ab-R99E mutant is severely affected in oligomerization that correlated with loss of toxicity^22^. To determine if the E101K mutation affected Cry1Ab oligomer formation, the purified trypsin-activated mutant toxin was incubated with a purified *M. sexta* CAD fragment for 30 min as previously reported^12,22^, samples were heated for 3 min at 25 °C, 50 °C or 100 °C before running the samples on SDS-PAGE electrophoresis since it was previously found that oligomeric structures of Cry1Ab are heat sensitive but SDS-resistant^12^. The resulting oligomeric structures were observed by western blotting using an anti-Cry1Ab polyclonal antibody. Figure 5 shows that the Cry1Ab-E101K mutant was severely affected in oligomer formation similar to mutant Cry1Ab-R99E, in contrast with Cry1Ab toxin that produced oligomeric structures of 180–200 kDa.

### Effects of reversed charge mutagenesis

To determine if the interaction between R99 and E101 in Cry1Ab or between E83 and R85 in Cry1Fa or D129 and K131 in Cry5Ba could be involved in a salt bridge formation, double reversed-charge mutations of these residues were constructed. Interestingly, the Cry1Ab-R99E-E101R double-point mutant partially recovered toxicity to *M. sexta* and *P. xylostella* larvae compared to the single Cry1Ab-R99E or Cry1Ab-E101K mutants (Table 1). Oligomerization assays showed that Cry1Ab-R99E-E101R was able to form oligomeric structures from activated toxin, although oligomer yields were lower compared to Cry1Ab wild type toxin correlating with the lower mortality induced by the double mutant when compared with the wild type toxin (Fig. 5). These data indicated that the correct position of the charges of R99 and E101 is necessary for the efficient toxicity of this toxin. Similarly, the double Cry1Fa-E83R-R85E mutant showed improved insecticidal activity against both lepidopteran larvae when compared with the single mutants (Table 1). In contrast, the Cry5Ba double E129K-K131D mutant did not recover toxicity to *C. elegans*.

### Introduction of an additional salt bridge in Cry1Ab-R99E

It is noteworthy that the effects on toxicity were more evident after mutagenesis of Cry1Ab toxin than the Cry1Fa toxin indicating that other important interactions must exist in stabilizing oligomers of Cry1Fa. To further characterize the role of the putative salt bridge that is present in Cry1Fa, we introduced this additional salt bridge into the non-toxic Cry1Ab-R99E mutant, in the same position as in Cry1Fa. Two mutagenesis strategies were followed to introduce this salt bridge in Cry1Ab, a triple mutant Cry1Ab-R99E-N85D-R87K or a double mutant Cry1Ab-R99E-N85E to have either D-K or R-E salt bridges in this position. However, Cry1AbR99E-N85E was highly unstable after treatment with trypsin and was not further characterized (Fig. 4B). Cry1Ab-R99E-N85D-R87K was stable and produced the expected 60 kDa activated toxin after treatment with trypsin (Fig. 4B). The bioassay data showed that Cry1Ab-R99E-N85D-R87K partially recovered toxicity to *P. xylostella* and *M. sexta* larvae in comparison to Cry1Ab (Table 1). The control protein Cry1Ab-N85D-R87K showed a similar toxicity as the wild type Cry1Ab, indicating that introduction of this putative salt bridge into the wild type toxin did not affect its activity (Table S1). Figure 5 shows that the Cry1Ab-R99E-N85D-R87K mutant partially recovered oligomerization, correlating with the toxicity of this triple mutant when compared to Cry1Ab toxin.

Introduction of double salt bridges into Cry1Ab (Cry1Ab-N85D-R87K) and Cry1Fa (Cry1Fa-T97D-R99K and Cry1Fa-T97E) did not increase or affect their toxicity against *P. xylostella* or *M. sexta* larvae when compared with the corresponding wild type toxins (Table S1). These Cry1Ab or Cry1Fa mutants were also tested against the lepidopteran *Spodoptera frugiperda*, which shows low susceptibility to Cry1Ab. Bioassay results showed similar toxicity as Cry1Ab or Cry1Fa wild-type toxins (data not shown), indicating that introduction of an additional salt bridge does not improve the toxicity of these toxins.

## Discussion

Electrostatic interactions are of fundamental importance in protein interactions. In the case of other pore-forming toxins such as lysteriolisin O toxin produced by *Listeria monocytogenes*, point mutations in charged residues of domain I α-helices that potentially form salt bridges in the interface of monomers resulted in toxins affected in oligomer formation and in hemolytic activity^35^. Also, in the pneumolysin toxin produced by *Streptococcus pneumoniae*, substitutions of K18 and R208 by alanine, disrupted the salt bridges that these residues form with E84 and D93 from adjacent monomers, respectively, and resulted in inactive cytolytic toxins^36^. These data highlight the critical role of specific electrostatic interactions involved in the assembly of monomers during oligomerization of different pore-forming toxins.

We analyzed the role in toxicity of putative salt bridges within two α-helices from adjacent monomers of different 3d-Cry toxins. The strategy was to compare the effect of single mutations that destroy the salt bridge with double reciprocal mutations that potentially restore the phenotype. Previously it was shown that an intramolecular salt bridge within the helix α-3 of the Cry4Ba toxin was important to maintain the correct structure of this toxin for oligomerization and insecticidal activity^32^. Based on a Cry1Ab structural model (Fig. 1), we predicted that E101 interacts with R99 from an adjacent monomer. Here we show that Cry1Ab domain I helix α-3 E101 was severely affected in toxin oligomerization and toxicity such as Cry1Ab-R99E mutant^22^, supporting that oligomerization is a key step in the Cry1Ab mode of action. Accordingly, Cry1Ab-R99E-E101R double-point mutant partially recovered oligomer formation and toxicity compared to single-point Cry1Ab-R99E or Cry1Ab-E101K mutants. The lower toxicity of Cry1Ab-R99E-E101R mutant compared to Cry1Ab (Table 1) correlated with the lower yield in oligomer formation (Fig. 5). However, it is remarkable that the double mutation recovered toxicity against *P. xylostella* and *M. sexta* showing at least 61-fold and 36-fold higher activity against these insects, when compared with either of the two single mutations (Cry1AbR99E and Cry1Ab-E101R) (Table 1). These data indicate that these residues contribute to the assembly of the functional oligomers that result in pore formation and the death of the larvae. The interactions between two monomers likely involve multiple contacts among the adjacent proteins, thus it was not unexpected that the recovery of toxicity of the reciprocal double mutant was not complete. The fact that the recovery of toxicity was partial, indicates that the original salt bridge was optimized to have the correct orientation of the proteins that would allow other contacts important for the following steps, such as membrane insertion and pore formation. Other examples showing partial restoration of protein function by reversing the charges of putative salt bridges include the neutralizing activity of monoclonal antibodies that block the activity of snake venoms towards the nicotinic acetylcholine receptor, where single-point mutants D31 in the antibody and R33 in the toxin affected their interaction and the double-point mutant with reversed charges partially restored this interaction^37^. Similarly, the interaction of trans-membrane domains in the cytoplasmic side of the G-protein-coupled receptors was demonstrated by single-point mutagenesis that affected protein activity and the function was only partially rescued in the double-point reversed charged mutant^38^.

Amino acid sequence alignment of the 3d-Cry toxin family of proteins showed that the salt bridge corresponding to Cry1AbR99-E101 is conserved in part of 3d-Cry toxin family. We analyzed a total of 91 sequences of the 3d-Cry family including one representative of each type (with the subindex *a* in the third range of the nomenclature^2^) showing that this salt bridge is present in 24 proteins of the 91 sequences analyzed (Fig. 3A labeled in yellow). While this is indeed a significant proportion of members of this family, it nonetheless indicates that other residues must be important for oligomerization in the remaining 3d-Cry proteins. Interestingly, the crystal structure of Cry5Ba trimer, where R99-E101 salt bridge is not conserved, showed an additional salt bridge that participates in the interaction of two helices α-3 from adjacent monomers. This second putative salt bridge is present in 48 of 91 sequences analyzed including the Cry1Fa toxin in the region corresponding to the loop between α-2b and α-3, indicating that this salt-bridge could only be formed if this loop forms part of an extended helix α-3 as in Cry4Ba and Cry5Ba trimeric structures (Fig. 3A labeled in green). Sequence analysis of helix α–3 showed that nine toxins contain the two salt bridges described here. Figure 3B shows a representation of helix α–3 of Cry1Ea and Cry1Ha toxins that contain both potential salt bridges. Single mutants in Cry5Ba and Cry1Fa in the second salt bridge were constructed. In the case of Cry5Ba the single mutants completely lost toxicity, indicating the salt bridge observed in the trimer structure of Cry5Ba is important for toxicity. The single mutants in Cry1Fa in this salt bridge resulted in a significant reduction of their insecticidal activity. Interestingly, the double Cry1Fa reciprocal mutant recovered the insecticidal activity. These data indicate that this salt bridge is important for Cry1Fa toxicity. The recovery of toxicity in the double reversal mutants of Cry1Ab and Cry1Fa support the idea that these salt bridges are formed *in vivo* and are important for Cry-toxin action. In the case of Cry5Ba we could not observe restoration of toxicity in the double reversal mutant (Cry5Ba-D129K-K131D). This is likely due to the possibility that this double reversal mutant could have a substantial loss of potency as was observed for the Cry1Ab double reversal mutant. It is important to mention that in this case it was not possible to increase the concentration of the toxin in the bioassay since assays were performed with *E. coli* expressing cells using the highest concentration of bacterial cells. It will be important to further analyze other Cry toxins of this family of proteins to confirm the participation of these salt bridges in toxicity.

In order to obtain additional evidence of the role of the structural change in the loop region between helices α-2b and α-3 in Cry1Ab, we introduced the second salt bridge into the Cry1Ab-R99E mutant toxin (Cry1Ab-R99E-N85D-R87K). Our results showed that this triple mutant resulted in an improved toxin showing 46-fold higher toxicity against *P. xylostella* and 40-fold higher toxicity against *M. sexta* when compared with the single non-toxic Cry1Ab-R99E mutant (Table 1), supporting that the loop region between α-2b and α-3 forms an extended α-helix 3 in Cry1Ab upon oligomerization (Fig. 1B). Thus, we propose that the second salt bridge stabilizes the extended helix α-3. Important conformational changes during toxin oligomerization and pore formation have been reported for other toxins such as the cytolysin A produced by *E. coli* and *Salmonella enterica* where the N-terminal helix flipped upwards and transitions of a β-sheet and loops into α-helical conformation were described^39^. Similarly, a transition of a loop and two α-helices into a β-sheet conformation was reported in the perfringolysin O toxin from *Clostridium perfringens*^40^.

Atomic force microscopy observations suggested that Cry1A toxin may be arranged as a tetrameric structure when it interacts with lipids^41^. However, Cry4Ba and Cry1AbMod toxin showed a trimeric array by electron microscopy observations^28,29^. The determination of the exact number of oligomer subunits of 3d-Cry toxins remains to be solved and is out of the scope of the present work. In any case, the salt bridges characterized here could be compatible with either a trimer or a tetramer structure of the toxin. Future work focused to solve the crystal structure conformation of both the pre-pore and the membrane inserted pore structures of 3d-Cry toxins will be important to verify the role of both salt bridges identified here and the conformation changes needed in 3d-Cry toxins for oligomerization and pore formation. The elucidation of such structures has primordial importance to understand the mechanism of action of 3d-Cry toxins.,

Finally, the partial restoration of toxicity and oligomerization in the Cry1Ab-R99E-N85D-R87K and the fact that some 3d-Cry toxins contains both salt bridges suggested that 3d-Cry toxicity could be improved by engineering salt-bridges between monomers in toxins where oligomerization could be a limiting step. Nevertheless, our results showed that the toxicity of Cry1Ab or Cry1Fa was not improved by adding an additional salt-bridge in these toxins (Table S1). It remains to be analyzed if toxicity could be improved by this strategy in other members of the 3d-Cry family. Overall our results support that helix α-3 plays a fundamental role in the process of oligomerization. Also, the fact that most members of 3d-Cry toxins contain the salt-bridge located in the loop region between α-2b and α-3 indicates that extension of helix α-3 maybe a necessary step for oligomerization and toxicity of the whole family of 3d-Cry toxins. This is the first report that reveals possible specific contacts among monomers that are important for toxicity and also indicates structural changes needed for oligomerization of 3d-Cry toxins *in vivo*. Moreover, the data show that a contact of different monomers of 3d-Cry toxins is a key step for larval toxicity.

## Materials and Methods

### Model construction of Cry1Ab trimer and in-silico analysis

A model of the trimeric structure of Cry1Ab toxin was constructed using Pymol (The PyMOL Molecular Graphics System, Version 1.8 Schrödinger, LLC https://www.pymol.org) performing structure alignments using the crystallographic trimer of Cry4Ba (PDB entry 1W99) as a template. Protein sequence alignments were done by Muscle 3.7 alignment^42^ using the amino acid sequences of 91 different Cry toxins. Identification of putative helix α-3 and orientation of the different faces if this helix was done by analyzing of the probability to adopt coiled coil conformation by using the program COILS that compares a protein sequence to data base of known coiled-coils and derives a similarity score^43,44^.

### Equilibrium MD simulations

The initial structure for the equilibrium simulations of the trimeric model of Cry1Ab was generated as described above, while that of the Cry5Ba trimer was from the crystal structure (PDB entry 4D8M)^11^. Since oligomerization experiments were performed at alkaline pH (pH 9–10.5), we first estimated the pKa of the titratable residues using Discovery Studio 3.5 (Dassault Systemes BIOVIA, San Diego, US) and protonated the residues as they would be at these high pH values. All trimers were solvated in TIP3 water in 0.15 M NaCl and minimized and equilibrated using VMD/NAMD and the CHARMM 27 force field^45–47^. The particle mesh Ewald algorithm was employed to treat electrostatic interactions, and the van der Waals interactions were treated with a cut-off of 12 Å. Langevin dynamics were employed to maintain a constant temperature of 310 K and a Nose-Hoover Langevin piston was used to maintain a constant pressure of 1 atm. The integration step was set to 2 fs. Extended simulations (80 ns) were performed with trimers of wild-type Cry1Ab, Cry1Ab-R99E, wild-type Cry5Ba, and Cry5Ba-D129K, with the mutations generated using VMD.

### Site directed mutagenesis

Plasmid pHT315-*cry1Ab* (constructed previously in our lab)^48^ containing wild type *cry*1Ab gene was used as template to construct single Cry1Ab mutants and plasmid pHT315-*Cry1AbR99E* (constructed previously in our lab)^22^ was used as template to make the double and triple Cry1Ab mutants by site directed mutagenesis. Mutagenesis was done using Quick-Change mutagenesis kit from Stratagene (La Jolla, CA) following the manufacturer’s instructions. The mutagenic oligonucleotides are described in Table 2. Plasmid pHT315-*cry1Fa* was constructed in this work by inserting *cry1Fa* gene (kindly provided by Dr. Jie Zhang, Institute of Plant Protection, Beijing, China) (GenBank AEH31417) into pHT315 vector (kindly provided by Dr. Didier Lereclus, Jouy-en-Josas INRA, France). Cry1Fa mutants were constructed by PCR using a homemade version of the protocol of mutagenesis. Briefly, a PCR reaction was performed with Phusion DNA polymerase, pHT315-*Cry1Fa* vector as DNA template and the mutagenic oligonucleotides described in Table 2. Plasmid pQE9-*cry5Ba* (kindly provided by Dr. Raffi Aroian from University of MA Medical School, USA) was used as template for site-directed mutagenesis of *cry5Ba* gene using Quick-Change mutagenesis kit as described above. Mutagenic oligonucleotides are described in Table 2.

**Table 2:** Mutagenic oligonucleotides.

Toxin	Mutated residues	Sequence
Cry1Ab	E101K	5′gga acc aag cca ttt cta gat taa agg gac taa gca atc 3′
Cry1Ab	R99E-E101R	5′cca agc cat ttc tga att acg cgg act aag caa tct tta tc 3′
Cry1Ab	N85D-R87K	5′gta caa att gaa cag tta att gac caa aaa ata gaa gaa ttc gct agg aac 3′
Cry1Ab	N85E	5′gta caa att gaa cag tta att gag caa aga ata gaa gaa ttc gct agg aac 3′
Cry1Fa	E83R	5′ctt tta cag att gaa caa ttg att cgg caa aga ata gaa aca ttg gaa agg aac cgg 3′
Cry1Fa	R85E	5′ctt tta cag att gaa caa ttg att gag caa gaa ata gaa aca ttg gaa agg aac cgg 3′
Cry1Fa	E83R/R85E	5′ctt tta cag att gaa caa ttg att cgg caa gaa ata gaa aca ttg gaa agg aac cgg 3′
Cry1Fa	T97D-R99K	5′gaa agg aac cgg gca att act gac tta aaa ggg tta gca gat agc tat g 3′
Cry1Fa	T97E	5′gaa agg aac cgg gca att act gaa tta cga ggg tta gca gat agc tatg 3′
Cry5Ba	D129K	5′gga tgc agt taa taa aat ggt aaa gaa taa gtt ctt aag tta taa tc 3′
Cry5Ba	K131D	5′cag tta ata aaa tgg tag ata atg att tct taa gtt ata atc tta gta c 3′
Cry5Ba	D129K-K131D	5′atg gat gca gtt aat aaa atg gta aag aat gat ttc tta agt tat aat ctt agt ac 3′

Plasmids contining *cry*1Ab or *cry*1Fa genes were transformed into *E. coli* DH5α cells. Automated DNA sequencing at Instituto de Biotecnología-UNAM facilities confirmed the point mutations. The mutated plasmids containing *cry1Ab* and *cry1Fa* genes were transformed into the acrystalliferous *B. thuringiensis* strain 407 as reported^49^. Transformant strains were selected in Luria broth at 30 °C supplemented with 10 μg ml^−1^ erythromycin and single colony lysates were used to amplify the *cry1Ab* or *cry1Fa* gene by PCR and confirm by DNA sequencing each of the mutant toxins expressed in Bt 407 strain. Plasmids containing *cry*5Ba genes were transformed into *E. coli* strain JM103.

### Purification Cry1Ab and Cry1Fa protoxins and activation

Nutrient broth sporulation medium (0.8% nutrient broth, 1 mM MgSO_4_.7H_2_O, 13 mM KCl, 10 mM MnCl_2_.4H_2_O, pH 7.0 supplemented with 2 ml/L of sterile solution of 131 mM FeSO_4_.7H_2_O in 1 N H_2_SO_4_ and 1 ml/L of sterile 0.5 M CaCl_2_) supplemented with erythromycin at 10 μg ml^−1^ was used for the expression of Cry1Ab or Cry1Fa or mutants proteins in Bt. After 3 days at 30 °C the sporulation process was completed and spores and crystals were harvested by centrifugation (10 min at 12,857 × *g*) and washed twice with 300 mM NaCl, 10 mM EDTA. The crystal inclusions were purified by discontinuous sucrose gradients^50^. Protoxins were solubilized in alkaline buffer: 50 mM Na_2_CO_3_, 0.2% β-mercaptoethanol, and pH 10.5 for 2 h and recovered after 20 min centrifugation at 12,857 × *g*. The pH of protoxin solution was lowered to pH 8.5 by adding 1: 4 (w/w) of 1 M Tris buffer pH 8.5 and soluble protoxin was activated with 1: 50 trypsin (trypsin: toxin) (TPCK treated trypsin from bovine pancreas, SIGMA Aldrich) for 2 h at 37 °C, Phenylmethylsulfonyl fluoride (PMSF)(1 mM final concentration) was added to stop proteolysis. Protein concentration was determined by the Bradford assay, using bovine serum albumin (SIGMA Aldrich) as standard.

The trypsin-activated toxins were loaded into a HiTrap Q HP column connected to the fast protein liquid chromatography system (ÄKTA, GE Healthcare Life Sciences). The column was washed with Buffer A (50 mM NaCl, 0.05 mM CO_3_/HCO_3_, pH 8.5) followed by elution with a gradient from 0–100% of Buffer B (1 M NaCl, 0.05 mM CO_3_/HCO_3_, pH 8.5). Finally, toxins were concentrated with Amicon® Ultra Centrifugal Filters (Millipore) and quantified as was described above.

*E. coli* JM103 transformed with the *cry5Ba* or mutated genes were grown at 37 °C in 2xYT/Amp 100 μg/ml to reach an OD_600_ of 0.6 and protoxin expression was induced with 1 mM isopropyl β-D-thiogalactoside for 16 h at 30 °C. Overnight cultures of *E. coli* strain JM103 carrying empty vector pQE9 or pQE9-*cry5Ba* were analyzed by SDS-PAGE and western-blot as described below to detect the Cry5Ba protoxin expression.

### Toxicity Assays against *P. xylostella* and *M. sexta* larvae

*M. sexta* larvae were reared for more than 15 years in our lab (from a colony kindly supplied by Dr. J. Ibarra CINVESTAV Irapuato México), *P. xylostella* larvae were provided by Benzon Research, USA. Bioassays were performed with *P. xylostella* 3^er^ instar and *M. sexta* neonate larvae using five to ten different concentrations of protoxin solutions that were poured on the surface of the diet. We used 24 well polystyrene plates, only one larvae was added per well and one plate per dose in triplicate. Mortality was analyzed after 7 days and the 50% lethal concentration (LC_50_) was calculated with Probit LeOra software. Negative controls without toxin addition were included in the bioassay.

### Toxicity against nematodes

Toxicity assays were based in the published protocol using synchronized L1- or L4-staged animals (nematode toxicity assays were kindly performed in Dr. Raffi Aroian laboratory from University of MA Medical School, USA)^34,51^. Each experiment was independently replicated.

### Expression and purification of a cadherin fragment

The *M. sexta*-CAD protein fragment (CR7-CR12) containing residues 810–1480 was expressed in *E. coli* ER2566 cells as previously reported^22^. The CAD fragment was purified using nickel affinity according to the manufacturer’s instructions (Qiagen, Germantown, MD).

### Analysis of Cry1Ab oligomerization

Oligomeric Cry1Ab structures were obtained by incubating 0.5 μg of the pure trypsin activated-toxin with the CAD fragment CR7-CR12 in a mass ratio 1: 4 (Cry1Ab: CAD-fragment) for 1 h at 37 °C in total volume of 100 μl of alkaline buffer in the presence of 0.5 μg of trypsin. Adding 1 mM PMSF stopped the reaction. Laemmli sample buffer 4x (0.125 M Tris/HCl, 4% SDS, 20% glycerol, 10% β-mercaptoethanol, 0.01% bromophenol blue) was added to the samples and they were divided into three tubes, incubated for five min at different temperatures (25 °C, 50 °C and 100 °C). These samples were separated in 8% SDS-PAGE and electro transferred to PVDF membranes (Millipore, Billerica, MA), which were used for western blot assays as described below.

### Western blot assays

The Cry1Ab polyclonal antibody was previously raised in our laboratory^52^. For western blot assays the PVDF membranes were blocked with 5% skimmed milk in PBS buffer pH 7.4 plus 0.1% Tween 20, for 1 h at room temperature. The membranes were rinsed once with same buffer. Cry1Ab toxin was detected after 1 h incubation with polyclonal anti-Cry1Ab (diluted 1/30,000) and then 1 h with goat anti-rabbit secondary antibody coupled to horseradish peroxidase (Santa Cruz) (diluted 1/20,000). Cry5Ba protoxin was detected with anti-6His HRP conjugated antibody (Qiagen) (diluted 1/5,000). Western blots were visualized by incubation with Super Signal chemiluminescence substrate (Pierce), according to the instructions of the manufacturer.

## Electronic supplementary material

Supplementary Information
