# Supplementary material for: Helix α-3 inter-molecular salt bridges and conformational changes are essential for toxicity of Bacillus thuringiensis 3D-Cry toxin family
Source: Sci Rep. 2018 Jul 9;8:10331. doi: 10.1038/s41598-018-28753-8 (PMC6037675; doi:10.1038/s41598-018-28753-8)

Helix α-3 inter-molecular salt bridges and conformational changes are essential for toxicity of *Bacillus thuringiensis* 3D-Cry toxin family

Sabino Pacheco[1], Isabel Gómez[1], Jorge Sánchez[1], Blanca-Ines García-Gómez[1], Daniel M. Czajkowsky[2], Jie Zhang[3], Mario Soberón[1] and Alejandra Bravo[1]

Supplementary Information

Table S1. Toxicity data of Cry1Ab or Cry1Fa mutants with double salt bridges against *Plutella xylostella* and *Manduca sexta* larvae.

Protein	*Plutella xylostella* [a]LC_{50} (fiducial limits)	*Manduca sexta* [a]LC_{50} (fiducial limits)
Cry1Ab	2.56 (0.79-3.79)	1.99 (1.61-2.42)
Cry1Ab-N85D-R87K	2.92 (2.42-3.47)	2.32 (1.99-2.76)
Cry1Fa	2.27 (0.65-3.56)	2.10 (1.75-2.51)
Cry1Fa-T97D-R99K	2.32 (1.91-2.77)	2.36 (1.58-2.99)
Cry1Fa-T97E	1.34 (0.44-1.97)	3.91 (3.34-4.63)

[a] LC_{50} value in ng toxin/cm^2 diet

Figure S1. Imagen of full-length blots shown in figure 5

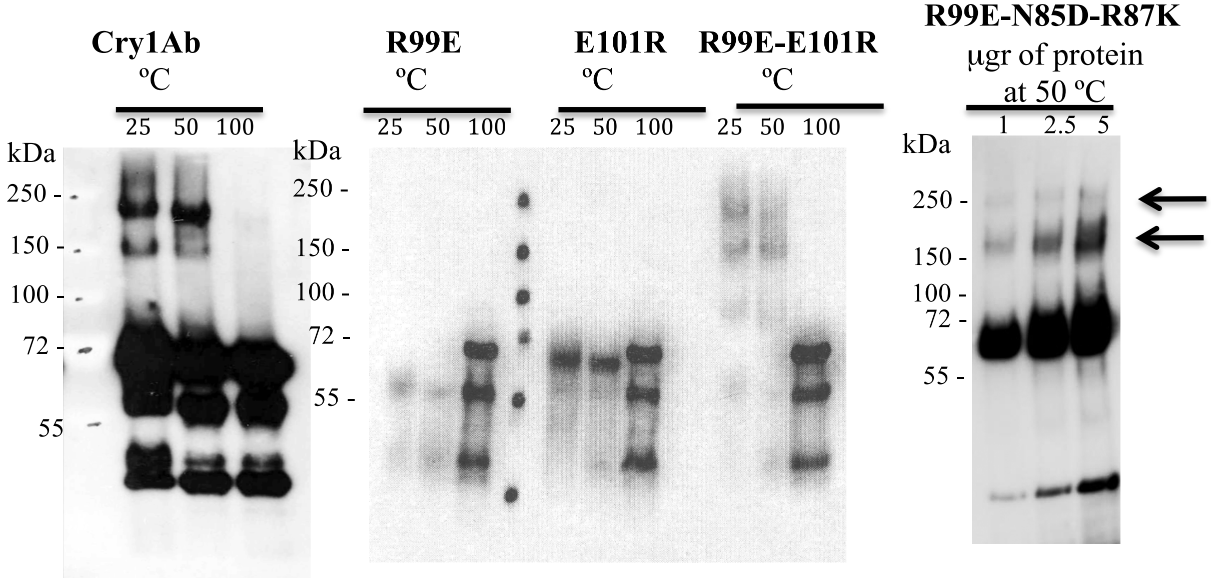

Supplement: Supplementary file 1 — Supplementary Information [file 41598_2018_28753_MOESM1_ESM.pdf]
